# Ethics that Fails to Regulate War, Ethics that Enhances War

**DOI:** 10.1007/s11673-024-10395-3

**Published:** 2025-01-09

**Authors:** Alphonso Lingis, Paul Komesaroff

**Affiliations:** 1 Pennsylvania State University Harrisburg, Middletown, PA USA; 2 Monash University, Melbourne, Australia

**Keywords:** War, Ethics

## Abstract

This short perspective piece argues that wars are often conducted in settings where ethical injunctions are ignored or overridden and where ethical oversight is avoided or circumvented. This is particularly the case with intrastate conflicts and is exacerbated by novel military technologies. In these and other settings ethics is often invoked actually to promote or prolong war.

## Editorial note

It is widely taken for granted that ethical discourse is a force for good because it invariably stands in opposition to violence, oppression, cruelty, and torture. But as the following note highlights, that is not the case: there are many circumstances in which arguments claiming to support ethical positions either turn a blind eye to evil or, worse, actually promote it. Furthermore, those responsible for such ethics-sanctioned atrocities are rarely, if ever, held accountable.

Sadly, in the modern world ethical support for injustice is not rare or even exceptional. Indeed, it has become a familiar fact of everyday life, to the extent that it is now even embedded in a culture that describes itself, without irony, as “humanitarian.” On all sides, the perpetrators of war and violence proclaim themselves champions of freedom, democracy, and peace. We may therefore legitimately wonder where, if anywhere, we can now turn to ground authentic opposition to the excesses from which we had once assumed ethics would protect us.

—Paul Komesaroff

## Ethical Injunctions That are Overridden

One hundred and ninety-six states ratified the four Geneva Conventions of 1949 establishing International Humanitarian Law. With the exception of what is now Ukraine, no developed Western nation has fought a war of national survival since 1945. They have fought wars to subjugate dissident states, to establish favourable geopolitical arrangements in crucial resource areas, to put an end to despotic regimes and to atrocities, and to suppress terrorism. They have devised cultural and psychological methods to win the “hearts and minds” of the enemy; they have perpetrated house-demolitions, detention without trial, torture, mass incarceration, and collective punishment.

Warring countries issue justifications that function to override ethical judgements. A military action may be assigned a moral purpose. It may be compared with doing nothing. A more forceful military action is taken to have more positive results than a more restrained one. A military action may be framed in ethically neutral, technical, or euphemistic terms. Soldiers “pacify” a territory. Attacks are depicted as “surgical strikes,” and noncombatants killed and wounded are listed as “collateral damage.” Direct attacks on noncombatants are justified by the claim that the enemy was using them as human shields of their troops or equipment.^1^ Dehumanization of the adversary motivates ethical disengagement.

## Conflicts and Methods That are Not Subject to Ethical Purview

### Intrastate Conflicts

Since World War II, there have been 285 intrastate conflicts or civil wars. In 2024 there are currently fifty-six conflicts. They have become more international, with ninety-two countries involved in conflicts outside their borders.

International humanitarian law does not extend to intrastate conflicts. Each state is accorded the right to defend itself against internal enemies. Each state deals with internal resistance and rebellion according to its own laws and policies. Resisters who kill in armed combat are treated as murderers.

Combatants of a state who control a significant region of that state are not within the purview of international law—and likewise, combatants recruited from and operating in several states. The states affected regard them as common law criminals and terrorists.^2^ When captured they may be subjected to indefinite imprisonment without trial, torture, and execution.

The ethics of war that legitimizes the self-defence of states is in conflict with the ethics, of ancient origin, of the right of rebellion. Enlightenment philosophers John Locke and John Stuart Mill were the most prominent thinkers to formulate this right in modern times.

The American, French, Russian, and Iranian revolutions have affirmed the right and duty of a people to alter or abolish an oppressive government. The right to revolution is affirmed in the U.S. Declaration of Independence, and it is enshrined in the constitutions of thirty-five American states and of thirty-five nation-states in the American hemisphere.

### New Military Technologies

Instead of massive recruitment of troops and heavy ordnance, increasingly, military operations comprise drone attacks and small commando actions that penetrate foreign countries to eliminate enemy leaders or opponents.^3^ Drones are very cheaply produced and the small numbers of military personnel involved motivates states to increase the places and targets of attacks.

International humanitarian law holds soldiers on all sides in conflicts between states to be moral equals, without regard to the justice of their cause, risking being killed and having the right to kill or be killed. Such combatants are not subject to criminal accountability for their actions. This symmetry and the justification of killing based on it no longer exists where a technician releases a drone to kill in a distant land and goes home to his or her family at the end of the day.

Soldiers will be equipped with communication technology, sensors, and musculoskeletal support platforms. They will be replaced by autonomous weapons systems that use sensor suits and computer algorithms to identify a target and trigger weapons to destroy the target without human control, thus without ethical control.

Only a small proportion of families in the warring country have husbands and sons and wives and daughters at risk in a war. The citizenry will be less informed and less attentive to military operations in distant countries. The arcane operations of AI in launching war will be little understood by the citizenry. The citizenry will be more liable to assent to a rush to war.

## War That is Enhanced by Ethics

A state that for political, nationalist, ethical, religious, economic, or strategic goals launches a war alleges ethical justifications. It is the ethical justifications that makes war acceptable to and enlist popular support for war.

Warring states prolong war, sometimes long after the costs in human lives and the devastation suffered exceed the goals originally envisioned, when its citizenry no longer supports the war, when it becomes clear that the war is unwinnable, and when a negotiated cessation of hostilities would be taken as evidence of a state’s weakness and unreliability by the adversary state and others. A warring state that legitimizes its war as ethically just and characterizes its adversary as unjust or evil, has more reason to prolong the war and resist negotiated settlement of the disputed claims of both sides, for it is unjust to compromise with injustice and evil.

If just war is ethically good war, war to protect vulnerable populations from oppression and human rights abuses—“humanitarian war”—has come to be ethically sanctioned and required.

In humanitarian wars increasing numbers of people are maimed, traumatized, raped, tortured, displaced, and starved. Drones subject populations to terrorizing regimes of surveillance. As more and more combatants and noncombatants are killed, as destruction of cities and lands extend in the state attacked, such wars prolong themselves. In these cases, ethics actively promotes war.

## Footnotes

1 Mahatma Gandhi is credited with creating the concept of human shield as a tool of resistance. U.S. Army Cavalry Scout Scott Ewing, who served in Iraq in the 2005–2006 period, stated that U.S. troops would give candy to Iraqi children so they would stay around their vehicles, thereby making more likely that enemies would not attack them. (Iraq Veterans and Glantz 2008, 70–71).

2 Secessionist armed forces, who seek to establish an independent state, tend to comply with the rules of war, which argues for international recognition of their legitimacy.

3 By the conclusion of the United States’ involvement in 1973, over 3.1 million Americans had been stationed in Vietnam.
